# Abnormal BMI in Male and/or Female Partners Are Deleterious for Embryonic Development and Pregnancy Outcome During ART Process: A Retrospective Study

**DOI:** 10.3389/fendo.2022.856667

**Published:** 2022-04-21

**Authors:** Lin Qi, Ya-ping Liu, Shi-ming Wang, Hao Shi, Xiao-li Chen, Ning-ning Wang, Ying-chun Su

**Affiliations:** Department of Center for Reproductive Medicine, The First Affiliated Hospital of Zhengzhou University, Zhengzhou, China

**Keywords:** BMI, high-quality embryo rate, clinical pregnancy rate, abortion rate, live birth rate

## Abstract

**Objective:**

To investigate the effect of BMI in male and/or female partners on embryo development and clinical pregnancy outcome during ART.

**Methods:**

Data of 11,130 cycles between January 2018 and December 2020 were retrospectively analyzed. They were divided into Group A, B, C, and D based on couples’ BMI values, also were divided into Group I, II, III and IV in IVF cycles and Group i, ii, iii, and iv in ICSI cycles. After grouping, inter-group indicators comparisons and logistic regression analysis were performed.

**Results:**

In IVF cycles, CPR in Group I and Group III were higher than Group IV. In Group III, it was higher than Group II. The AR in Group IV was higher, but the LBR was lower than Group I, Group II, and Group III. Logistic regression analysis results suggested that AR in Group IV was higher than that in Group I in IVF cycles, whereas LBR was lower.

In ICSI cycles, high-quality embryo rate in Group i and Group ii were both higher than that in Group iii and Group iv. The CPR in Group i was higher than Group ii and Group iv, and in Group iii was higher than Group ii and Group iv. The AR in Group i was lower than Group iii and Group iv, and AR in Group ii was lower than Group iv. LBR, in Group I it was higher than Group ii, Group iii, and Group iv. Logistic regression analysis results suggested CPR in Group ii was significantly lower than that in Group i. AR in Group iii was considerably higher than that in Group i. LBR in Group ii and Group iv were significantly lower than that in Group i.

**Conclusion:**

Female higher BMI was not conducive to the formation of high-quality embryos in ICSI cycle. Female and/or male BMI affected AR and LBR more than CPR not only in IVF cycles, but also in ICSI cycles.

## Introduction

Overweight and obesity, which have already reached epidemic levels in some countries, have become topics of urgent importance. The World Health Organization (WHO) defined overweight as a body mass index (BMI) of over 25 kg/m²; a BMI over 30 kg/m² is considered to indicate obesity (1). WHO data for 2016 showed that 13% of the world’s adults were obese, with obesity prevalence of 15% in women and 11% in men. Obesity is an important risk factor for a number of non-communicable diseases, such as hypertension, cardiovascular disease, type 2 diabetes and other metabolic diseases (2), osteoarthritis (3), gallstone disease (4), asthma, and other chronic respiratory diseases (5–7), and multiple malignancies (8).

Obesity is often accompanied by changes in the levels of endocrine and reproductive hormones. Overweight women are more likely to suffer from polycystic ovary syndrome, menstrual disorders, infertility, miscarriage, poor pregnancy outcomes, and multiple pregnancy complications (including gestational diabetes, pre-eclampsia, and fetal macrosomia) (9–12). Studies have shown that high female BMI is associated with reduced blastocyst formation rate during *in vitro* fertilization (IVF) (13), abnormal oocyte morphology (14), low clinical pregnancy rate (CPR), high abortion rate (AR), and low live birth rate (LBR) (15–23). However, other research findings also suggested that female BMI had no influence on the assisted reproduction technology (ART) outcomes (24–28). This may be due to differences in the distribution of people selected in their study, as well as in the BMI grouping.

Meanwhile, male BMI has been related to sperm parameters. In men, obesity was found to cause low sperm concentration, low percentage of forward-moving sperm, low rate of normal sperm morphology, and high DNA fragmentation index (DFI%) (29, 30). Nevertheless, Anifandis et al. suggested that male BMI was not related to sperm parameters (31, 32), but it did affect embryo quality, and led to bad pregnancy outcomes (20, 27, 32–35). Conversely, the results of other studies suggested that BMI did not affect *in vitro* fertilization, embryo development and quality, pregnancy outcomes, and obstetric outcomes (24, 30, 31, 33, 36–38).

However, these studies have rarely considered the effect of BMI on oocytes in women and sperm quality men. Meanwhile, comprehensive and holistic assessments of ART pregnancy outcomes have been rarely conducted. Therefore, the aim of this study was to evaluate the effects of female and male BMI on the process of ART. In addition, most of the previous investigations have been focused on determining the impact of BMI and ART on pregnancy outcomes in women, but little attention has been paid to that influence on ART in men. Therefore, here, our purpose was to assess the overall impact of BMI of female and male subjects on *in vitro* fertilization, embryo development, and clinical outcome during the first cycle of ovulation induction to provide improved guidance that would increase clinical ART pregnancy success rates.

## Materials and Methods

### Patients

We collected the data of 24,081 cycles of patients that had undergone *in vitro* fertilization treatment in the First Affiliated Hospital of Zhengzhou University, the Reproductive and Genetic Hospital between January 2018 and December 2020. Based on the inclusion and exclusion criteria, we eventually analyzed the clinical data of 11,130 cycles.

Based on the BMI guidelines for the Chinese population (17, 39), the couples were divided into four groups based on the couple’s BMI. They were divided into the following groups: Group A (female and male BMI < 24 kg/m², N = 2526); Group B (female BMI < 24 kg/m² and male BMI ≥ 24 kg/m², N = 4349); Group C (female BMI ≥ 24 kg/m² and male BMI < 24 kg/m², N = 1455); and Group D (female and male BMI ≥ 24 kg/m², N = 2800). Then, the baseline data of each group were compared with those of the other groups and analyzed. Next, the data of these groups were divided into IVF and ICSI groups depending on the method of insemination. Further, BMI = 24 kg/m² was set as the threshold value, based on the results of the previous analysis. IVF cycles were divided into Group I (female and male BMI < 24 kg/m², N = 2007); Group II (female BMI < 24 kg/m² and male BMI≥ 24 kg/m², N = 3483); Group III (female BMI ≥ 24 kg/m² and male BMI< 24 kg/m², N = 1179); and Group IV (female and male BMI ≥ 24 kg/m², N = 2275). Meanwhile, ICSI cycles data were divided into Group i (female and male BMI < 24 kg/m², N = 519); Group ii (female BMI < 24 kg/m² and male BMI ≥ 24 kg/m², N = 866); Group iii (female BMI ≥ 24 kg/m² and male BMI< 24 kg/m², N = 276); and Group iv (female and male BMI ≥ 24 kg/m², N = 525). After grouping, the indicators of embryo development and pregnancy outcomes were compared among the groups, including oocyte maturation rate, normal fertilization rate, cleavage rate, high-quality embryo rate, blastocyst formation rate, high score blastocyst rate, CPR (number of pregnancy cycles/number of transplant cycles), AR (number of abortion cycles/number of clinical pregnancy cycles) and LBR (number of live birth cycles/number of transplant cycles).

**Figure 1:**
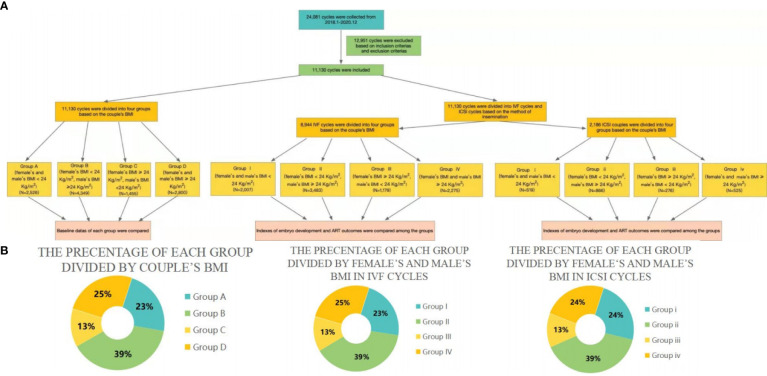
**(A)** Flow charts representing the populations included in the current study. **(B)** Pie charts representing the percentages of different groups.

AR, and LBR. A diagrammatic representation of our study is depicted in **Figure 1A**; the percentages of the different groups are displayed in **Figure 1B**).

### Inclusion Criteria and Exclusion Criteria

The following inclusion criteria were applied: (1) Couples undergoing *in vitro* fertilization-embryo transfer (IVF-ET) or intracytoplasmic sperm injection-embryo transfer (ICSI-ET) in the first cycle; (2) Couples with successful egg retrieval, embryo formation, and fresh transplantation after controlled ovarian stimulation (COS); (3) Men and women with complete BMI and follow-up records; (4) Female’s and male’s BMI ≥18.5 kg/m².

The exclusion criteria were as follows: (1) Donor and frozen semen cycles; (2) Preimplantation genetic diagnosis/screening (PGD/PGS) cycles; (3) Intrauterine insemination cycles; (4) Cycles of incomplete follow-up data; (5) Women and/or men with chromosome abnormality or (and) genetic disorder; (6) Men with diseases that affect semen quality or lead to infertility, such as urinary tract and genital infections, and medications.

### Analysis of Semen, DFI% Detection, Treatment of the Semen, and *In Vitro* Fertilization-Embryo Formation

Semen was routinely analyzed by marker plate count and hematoxylin-eosin (HE) staining. The volume of the semen obtained, sperm concentration, percentage of progressive motility (PR) sperm, percentage of non-progressive motility (NP) sperm, percentage of in-motility (IM) sperm, and DFI% were measured. DFI% was detected by sperm chromatin structure analysis (SCSA). The criteria to evaluate the sperm parameters based on the 5^th^ World Health Organization guidelines (40). The male spouse ejaculated once 2–7 days before oocyte retrieval and again on the day of oocyte retrieval. At room temperature, the semen was treated with density gradient centrifugation and upstream method to remove impurities and abnormal sperm from seminal plasma and semen and capacitate the sperm. The oocytes were collected 37 hours after HCG injection, the granulosa cells were removed after short-term fertilization, and the prokaryocyte formation was observed 16–18 hours after fertilization. Vitrolife (Västra Frölunda, Sweden) or COOK medium was used for *in vitro* culture.

### Embryo Transplantation and Luteal Support

High-quality D3 cleavage-stage or D5 blastocyst-stage embryos were transplanted on the 3^rd^ or 5^th^ day after oocyte retrieval, based on the morphological evaluation of the embryos and the physical condition of the woman patients. The grading criteria of high-quality embryos were based on previous of our publications center (41). We used 1–2 cleavage-stage embryos or 1 blastocyst-stage embryos for transplantation. Surplus embryos or blastocysts were selected according to certain quality standards, and the surplus embryos or blastocysts were vitrified. Luteal phase support was given on the day of egg retrieval.

### Regular Follow-Up Examinations

The serum chorionic gonadotropin (β-HCG) levels were measured 14/18 days after the embryo transfer. A threshold of β-HCG > 50 IU/L was set for biochemical pregnancy. Abdominal color Doppler ultrasound was performed 35 days after the embryo transfer to determine whether a gestational sac had been formed. An ultrasound finding of a gestational sac was considered to indicate a clinical pregnancy (42). Then, the patients were followed up regularly by telephone to obtain information on their periodic coverage, pregnancy, and obstetric outcomes (such as live births and third-trimester abortions), which were recorded in the central archives.

### Statistical Analysis

SPSS 25.0 software was employed for data analysis. Continuous numerical variables were represented by mean ± SD. One-way ANOVA and Mann-Whitney U-test were used to analyze the differences among groups. Categorical variables were represented by frequency and percentage (%), and the differences between groups were assessed by chi-square test (χ^2^-test). Logistic regression was used to analyze the data and explore the factors affecting the CPR, AR and LBR. The binary outcome of this model was whether clinical pregnancy, abortion and live birth or not. P < 0.05 was considered to indicate statistically significant differences.

## Results

We collected clinical data from 11,130 infertile couples who received assisted reproductive technology treatment at our center between January 2018 and December 2020. They received fresh transplants, and were followed up. Initially, the couples were divided into four groups (Group A, B, C, and D) based on the couple’s BMI. Then, we analyzed their baseline data and conducted comparison among the four groups. The demographic characteristics, baseline endocrine levels, and other data of the female patients in the different subgroups are presented as mean ± SD in **Table 1**.

**Table 1a:** Baseline data of female and male in different groups.

Item	Group A (Female and Male BMI < 24 kg/m^2^, N=2,526)	Group B (Female BMI < 24 kg/m^2^ and Male BMI≥ kg/m^2^, N=4,349)	Group C (Female BMI ≥ 24 kg/m^2^ and Male BMI< kg/m^2^, N=1,455)	Group D (Female and Male BMI ≥ kg/m^2^, N=2,800)	P value
Female					
Age (year)	30.55±4.60	31.61±4.81	30.77±5.04	32.00±5.13	<0.01^a^ ^c^ ^αβγ^
Basal FSH (mIU/mL)	7.09±2.53	7.11±2.70	6.51±2.18	6.65±2.36	<0.030^b^ ^c^ ^αβ^
Basal LH (mIU/mL)	5.86±4.23	5.83±4.62	5.93±4.79	5.50±4.82	0.027^β^
Basal AMH (mIU/mL)	3.53±2.80	3.32±2.70	3.92±3.26	3.64±3.12	<0.05 ^a^ ^b^ ^αβγ^
AFC	14.14±6.39	13.47±6.52	15.57±7.07	14.84±7.26	<0.010^a^ ^b^ ^c^ ^αβγ^
No. of oocytes retrieved	12.89±5.74	12.36±5.80	13.26±6.18	12.79±6.28	<0.02^^a^αβ^
No. of MII oocytes	10.61±5.02	10.18±5.02	10.87±5.31	10.53±5.42	<0.02^a^ ^α^
Male					
Age (year)	31.32±5.38	32.56±5.61	31.42±5.37	32.82±5.67	0.000^a^ ^c^ ^αβ^
Semen volume (ml)	3.07±1.35	3.01±1.23	3.02±1.26	3.00±1.33	<0.010^a^ ^c^
Concentration (10^6^/ml)	46.24±34.05	45.35±36.34	46.44±34.68	45.15 ±35.02	>0.05^a^ ^b^ ^c^ ^αβγ^
PR (%)	32.65±13.27	31.75±14.01	32.49±12.93	31.85±13.32	<0.05^a^ ^c^ ^α^
NP (%)	9.11±3.92	9.41±3.78	9.32±4.07	9.37±3.65	<0.010^a^ ^c^
IM (%)	51.89±18.52	53.21±17.45	52.61±16.95	53.25±16.54	<0.010^a^ ^c^
DFI (%)	13.60±7.37	16.44±10.71	13.58±7.46	17.42±11.47	0.000^a^ ^c^ ^αγ^

BMI, Body mass index; FSH, follicle stimulating hormone; LH, luteinizing Hormone; AMH, Anti-Müllerian hormone; AFC, Antral Follicle Count; PR, Progressive motility; NP, Non-progressive motility; IM, In-motility; DFI, Sperm DNA fragmentation index. P < 0.05 was considered to be statistically significant.

a Group A vs Group B.

b Group A vs Group C.

c Group A vs Group D.

^α^Group B vs Group C.

^β^Group B vs Group D.

^γ^Group C vs Group D.

**Table 1b:** Etiological classification and methods of assisting pregnancy in different groups.

Item	Group A (Female and Male BMI < 24 kg/m^2^, N=2,526)	Group B (Female BMI < 24 kg/m^2^ and Male BMI≥ kg/m^2^, N=4,349)	Group C (Female BMI ≥ 24 kg/m^2^ and Male BMI< 24 kg/m^2^, N=1,455)	Group D (Female and Male BMI ≥ 24 kg/m^2^, N=2,800)	P value
Etiology					0.000^abcαβγ^
Tubal factors	49.47%(1249/2525)	46.65% (2029/4349)	43.64% (635/1455)	42.82% (1199/2800)	
Endometriosis	6.22% (157/2525)	6.62% (288/4349)	4.26% (62/1455)	4.21% (118/2800)	
PCOS	6.50% (164/2525)	6.25% (272/4349)	15.60% (227/1455)	14.50% (406/2800)	
Ovarian disease	3.72% (94/2525)	4.48% (195/4349)	4.12% (60/1455)	5.39% (151/2800)	
For unknown reasons	25.11% (634/2525)	28.01% (1218/4349)	24.95% (363/1455)	25.14% (704/2800)	
Male factors	8.99% (227/2525)	7.98% (347/4349)	7.42% (108/1455)	7.96% (223/2800)	
Method of ART					0.368^abcαβγ^
IVF	79.49% (2007/2525)	80.01% (3483/4349)	81.03% (1179/1455)	81.25% (2275/2800)	
ICSI	20.51% (518/2525)	19.91% (866/4349)	18.97% (276/1455)	18.75% (526/2800)	

BMI, Body mass index; PCOS, Polycystic ovary syndrome; ART, assisted reproduction technology; IVF, in vitro fertilization; ICSI, intracytoplasmic sperm injection. P < 0.05 was considered to be statistically significant.

a Group A vs Group B.

b Group A vs Group C.

c Group A vs Group D.

^α^Group B vs Group C.

^β^Group B vs Group D.

^γ^Group C vs Group D.

Male factors include oligozoospermia, asthenospermia, teratozoospermia, dysspermia, and chromosome and single gene genetic disorders, and so on.

As can be seen in **Table 1A**, there were significant differences in female age, basal hormone level, antral Follicle Count (AFC), DFI%, No. of oocytes retrieved and MII among the groups. And male age, semen volume, progressive motility (PR), non-progressive motility (NR), in-motility (IM) and DFI% among the groups also were significantly different. Differences among the four groups, were also established in the age of females (P < 0.01), but there was no difference except for that between Group C and group D (P > 0.05). Group A was constituted by the youngest participants, whereas Group D included the oldest. There was a similar pattern of male age distribution among the four groups (P < 0.001), but there was also no difference except between Group C and Group D (P > 0.05). No difference was found in the basal FSH levels between Group A and Group B and between Group C and Group D (P > 0.05). However, the basal FSH levels in Group A and Group B were significantly higher than those in Group C and Group B (P < 0.001). As visible in **Table 1**, the basal LH level of Group A and Group B were higher than that of Group D (P < 0.05). Nevertheless, there was no significant difference between the other groups (P > 0.05). Except for Group A and Group D (P > 0.05), there were differences in basal AMH levels among groups (P < 0.05). Group C had the highest basal AMH level, whereas Group B had the lowest. Significant differences in AFC were detected among the four groups (P < 0.01). The No. of oocytes retrieved in Group B was significantly lower than those in Group A, Group C, and Group D (P < 0.05). No significant differences were observed among the other groups (P > 0.05). Except for Group A and Group B, Group B and Group C, there was no difference in No. of MII oocytes between the other groups (P > 0.05). The No. of MII oocytes in Group B was significantly lower than those in Group A and Group C (P < 0.05). Compared with Group A, semen volume and PR in Group B and Group D were obviously lower (P < 0.05). While NP, IM and DFI% in Group B and Group D were significantly higher than that in Group A (P < 0.05). PR in Group C was higher than that in Group B, while DFI% in Group C was lower (P < 0.05). The same as DFI% in Group C was lower than that in Group D (P < 0.05).

**Table 1B** showed that the distribution of causes of infertility was different among groups, but the method of fertilization was the different. There were differences in the causes of infertility among the four groups (P < 0.001). Among the four groups, the proportions of PCOS in Group C (15.60%) and Group D (14.50%) were significantly higher than those in Group A (6.50%) and Group B (6.25%).

**Table 2** presents the effects of female’s BMI on oocytes maturation, female’s and (or) male’s BMI oocytes fertilization, and embryo development in IVF cycles (**Table 2A**) and ICSI cycles (**Table 2B**). As seen in **Table 2A**, Group II had a higher 2PN fertilization rate than Group III (0.66 ± 0.19 vs 0.64 ± 0.18, p = 0.006). However, no differences were found in the mature oocyte rate, 2PN fertilization rate, normal fertilization cleavage rate, high-quality embryo rate, blastocyst formation, and high-scoring blastocyst rate (P > 0.05). As can be observed in **Table 2B**, there was no difference in the mature oocyte rate, 2PN fertilization rate, normal fertilization cleavage rate, blastocyst formation, and high-scoring blastocyst rate (P > 0.05). The high-quality embryo rates of Group i (0.69 ± 0.25) and Group ii (0.69 ± 0.25) were higher than those of Group iii (0.64 ± 0.28) and Group iv (0.62 ± 0.22) (P < 0.05).

**Table 2a:** Effect of female or (and) male BMI on oocytes maturation, fertilization, embryo development, and embryo quality in IVF cycles.

Item	Group I (Female and Male BMI < 24 kg/m^22^, N=2,007)	Group II (Female BMI < 24 kg/m^2^ and Male BMI≥ 24 kg/m^2^, N=3,483)	Group III (Female BMI ≥ 24 kg/m^2^ and Male BMI< 24 kg/m^2^, N=1,179)	Group IV (Female and Male BMI ≥ kg/m^2^, N=2,275)	P value
Mature oocyte rate (%)	0.82±0.15	0.83±0.15	0.83±0.15	0.83±0.16	>0.05^a^ ^b^ ^c^ ^αβγ^
2PN fertilization rate (%)	0.65±0.18	0.66±0.19	0.64±0.18	0.65±0.19	0.006^α^ >0.05^a^ ^b^ ^c^ ^βγ^
Normal fertilization cleavage rate (%)	0.99±0.05	0.99±0.05	0.99±0.04	0.99±0.05	>0.05^a^ ^b^ ^c^ ^αβγ^
High quality embryo rate (%)	0.71±0.25	0.70±0.25	0.71±0.27	0.72±0.25	>0.05^a^ ^b^ ^c^ ^αβγ^
Blastocyst formation rate (%)	0.52±0.33	0.51±0.34	0.52±0.32	0.51±0.33	>0.05^a^ ^b^ ^c^ ^αβγ^
High scoring blastocyst rate (%)	0.29±0.32	0.28±0.32	0.27±0.30	0.28±0.32	>0.05^a^ ^b^ ^c^ ^αβγ^

BMI, Body Mass Index; 2PN, two Pronuclear; P < 0.05 was considered to be statistically significant.

a Group I vs Group II.

b Group I vs Group III.

c Group I vs Group IV.

^α^Group II vs Group III.

^β^Group II vs Group IV.

^γ^Group III vs Group IV.

**Table 2b:** Effect of female and Male BMI on oocytes maturation, fertilization, embryo development, and embryo quality in ICSI cycles.

Item	Group i (Female and Male BMI < 24 kg/m^2^, N=519)	Group ii (Female BMI < 24 kg/m^2^ and Male BMI ≥ 24 kg/m^2,^ N=866)	Group iii (Female BMI ≥ 24 kg/m^2^ and Male BMI < 24 kg/m^2^, N=276)	Group iv (Female and Male BMI ≥ 24 kg/m^2^, N=525)	P value
Mature oocyte rate (%)	0.85±0.14	0.84±0.14	0.84±0.14	0.84±0.14	>0.05^a^ ^b^ ^c^ ^αβγ^
2PN fertilization rate (%)	0.75±0.18	0.75±0.18	0.74±0.21	0.75±0.19	>0.05^a^ ^b^ ^c^ ^αβγ^
Normal fertilization cleavage rate (%)	0.98±0.06	0.99±0.05	0.99±0.04	0.99±0.05	>0.05^a^ ^b^ ^c^ ^αβγ^
High quality embryo rate (%	0.69±0.25	0.69±0.25	0.64±0.28	0.62±0.22	0.02^b^ ^α^ 0.000^c^ ^β^ >0.05^a^ ^γ^
Blastocyst formation rate (%)	0.47±0.32	0.50±0.33	0.50±0.33	0.49±0.33	>0.05^a^ ^b^ ^c^ ^αβγ^
High scoring blastocyst rate (%)	0.24±0.30	0.26±0.31	0.26±0.31	0.28±0.31	>0.05^a^ ^b^ ^c^ ^αβγ^

BMI, Body Mass Index; 2PN, two Pronuclear; P < 0.05 was considered to be statistically significant.

a Group i vs Group ii.

b Group i vs Group iii.

c Group i vs Group iv.

^α^Group ii vs Group iii.

^β^Group ii vs Group iv.

^γ^Group iii vs Group iv.

Further, we analyzed the clinical pregnancy outcomes of each group (**Tables 3A**, **3B**).

**Table 3a:** Effect of female and Male BMI on pregnancy outcome in IVF cycles.

Item	Group I (Female and Male BMI < 24 kg/m^22^, N=2,007)	Group II (Female BMI < 24 kg/m^2^ and Male BMI ≥ 24 kg/m^2^, N=3,483)	Group III (Female BMI ≥ 24 kg/m^2^ and Male BMI < 24 kg/m^2^, N=1,179)	Group IV (Female and Male BMI ≥ kg/m^2^, N=2,275)	P value
CRP (%)	62.13% (1247/2007)	60.24% (2098/3483)	64.03% (755/1179)	59.12% (1345/2275)	0.045^c^ 0.021^α^ 0.005^γ^ >0.05^a^ ^b^ ^β^
AR (%)	13.95% (174/1247)	15.82% (332/2098)	15.63% (118/755)	20.00% (269/1345)	0.000^c^ 0.002^β^ 0.014^γ^ >0.05^a^ ^b^ ^α^
LBR (%)	53.46% (1073/2007)	50.70% (1766/3483)	54.03% (637/1179)	47.25% (1075/2275)	0.000^c^ ^γ^ 0.011^β^ >0.05^a^ ^b^ ^α^

BMI, Body Mass Index; CPR, clinical pregnancy rate; AR, abortion rate; LBR, live birth rate, P < 0.05 was considered to be statistically significant.

a Group I vs Group II.

b Group I vs Group III.

c Group I vs Group IV.

^α^Group II vs Group III.

^β^Group II vs Group IV.

^γ^Group III vs Group IV.

**Table 3b:** Effect of female and Male BMI on pregnancy outcome in ICSI cycles.

Item	Group i (Female and Male BMI < 24 kg/m^2^, N=519)	Group ii (Female BMI < 24 kg/m^2^ and Male BMI ≥ 24 kg/m^2,^ N=866)	Group iii (Female BMI ≥ 24 kg/m^2^ and Male BMI < 24 kg/m^2^, N=276)	Group iv (Female and Male BMI ≥ 24 kg/m^2^, N=525)	P value
CRP (%)	68.40% (357/519)	59.12% (512/866)	68.12% (188/276)	60.38% (317/525)	0.000^a^ 0.005^c^ 0.009^α^ 0.031^γ^ >0.05^b^ ^β^
AR (%)	8.68% (31/357)	11.72% (60/512)	19.15% (36/188)	14.83% (47/317)	0.001^b^ 0.016^c^ 0.013^α^ >0.05^a^ ^βγ^
LBR (%)	62.62% (325/519)	52.19% (452/866)	55.07% (152/276)	51.62% (271/525)	0.000^a^ ^c^ 0.040^b^ >0.05^αβγ^

BMI, Body Mass Index; CPR, clinical pregnancy rate; AR, abortion rate; LBR, live birth rate, P < 0.05 was considered to be statistically significant.

a Group i vs Group ii.

b Group i vs Group iii.

c Group i vs Group iv.

^α^Group ii vs Group iii.

^β^Group ii vs Group iv.

^γ^Group iii vs Group iv.

The results displayed in **Table 3A** showed that in the IVF cycles, the CPR in Group I and Group III were significantly higher (62.13% vs 59.12%, P = 0.045; 64.03% vs 59.12%, P = 0.005) than that in Group IV. The CPR in Group III was significantly higher than that in Group II (64.03% vs 60.24%, P = 0.021). However, no differences in CPR among the other groups (P > 0.05) were established. The AR in Group IV was higher than those in Group I, Group II, and Group III (20.00% vs 13.95%, P = 0.000; 20.00% vs 15.82%, P = 0.002; 20.00% vs 15.63%, P = 0.014). Additionally, the LBR in Group IV was lower than those in Group I, Group II, and Group III (47.25% vs 53.46%, P = 0.000; 47.25% vs 50.70%, P = 0.011; 47.25% vs 54.03%, P = 0.000). No differences in AR and LBR were detected among the other groups (P > 0.05).

The data obtained for the ICSI cycles showed that the CPR of Group i was significantly higher than those of Group ii and Group iv (68.40% vs 59.12%, P = 0.000; 68.40% vs 60.38%, P = 0.005); that of Group iii was significantly higher than those of Group ii and Group iv (68.12% vs 59.12%, P = 0.009; 68.12% vs 60.38%, P = 0.031). Nevertheless, there was no difference in CPR between Group i and Group iii and between Group ii and Group iv (**Table 3B**). At the same time, we analyzed the differences in AR and LBR in the ICSI cycles among the four groups. Group iii had significantly higher AR values than those in Group i and Group ii (19.15% vs 8.68%, P = 0.001; 19.15% vs 11.72%, P = 0.016). The LBR in Group i was significantly higher than those in Group ii, Group iii, and Group iv (62.62% vs 52.19%, P = 0.000; 62.62% vs 55.07%, P = 0.040, 62.62% vs 51.62%, P = 0.000). Nonetheless, the values of AR and LBR in the other groups were similar, with no difference among the groups (P > 0.05) (**Table 3B**).

To further investigate the effect of female and male BMI on the pregnancy outcomes, we excluded the effects of female’s and male’s age, infertility cause, basal FSH level, basal LH level, basal AMH level, AFC, No. of oocytes retrieved, No. of MII oocytes, and DFI% by logistic regression analysis; the results are shown in **Tables 4A**, **B**.

**Table 4a:** Logistic regression analysis on the effect of female and male BMI on pregnancy outcomes in IVF cycles.

Item	Group I (Female and Male BMI < 24 kg/m^2^)	Group II (Female BMI < 24 kg/m^2^ and Male BMI ≥ 24 kg/m^2^)	P value	Group III (Female BMI ≥ 24 kg/m^2^ and Male BMI < 24 kg/m^2^)	P value	Group IV (Female and Male BMI ≥ 24 kg/m^2^)	P value
CPR							
OR (95% CI)	Reference	0.92 (0.83-1.03)	0.165	1.09 (0.94-1.26)	0.283	0.88 (0.78-1.00)	**0.044**
aOR (95% CI)	Reference	1.01 (0.88-1.16)	0.894	1.12 (0.93-1.35)	0.233	0.97 (0.83-1.13)	0.671
AR							
OR (95% CI)	Reference	1.16 (0.95-1.41)	0.144	1.14 (0.89-1.47)	0.303	1.15 (1.25-1.90)	**0.000**
aOR (95% CI)	Reference	1.08 (0.88-1.32)	0.477	1.18 (0.91-1.53)	0.214	1.46 (1.17-1.81)	**0.000**
LBR							
OR (95% CI)	Reference	0.90 (0.80-0.99)	**0.049**	1.023 (0.89-1.18)	0.757	0.78 (0.69-0.88)	**0.000**
aOR (95% CI)	Reference	1.01 (0.90-1.14)	0.840	0.97 (0.84-1.13)	0.721	0.86 (0.75-0.97)	**0.017**

CPR, clinical pregnancy rate; AR, abortion rate; LBR, live birth rate; OR, Ratio ratio; aOR, Adjusted Ratio ratio; bold values means P < 0.05 was considered to be statistically significant.

**Table 4b:** Logistic regression analysis on the effect of female and male BMI on pregnancy outcomes in ICSI cycles.

Item	Group i (Female and Male BMI < 24 kg/m^2^)	Group ii (Female BMI < 24 kg/m^2^ and Male BMI ≥ 24 kg/m^2^)	P value	Group iii (Female BMI ≥ 24 kg/m^2^ and Male BMI < 24 kg/m^2^)	P value	Group iv (Female and Male BMI ≥ 24 kg/m^2^)	P value
CPR							
OR (95% CI)	Reference	0.66 (0.52-0.83)	**0.000**	0.97 (0.71-1.33)	0.860	0.70 (0.54-0.90)	**0.000**
aOR (95% CI)	Reference	0.73 (0.58-0.93)	**0.012**	1.03 (0.74-1.44)	0.868	0.79 (0.60-1.04)	0.097
AR							
OR (95% CI)	Reference	1.39 (0.88-2.20)	0.156	2.48 (1.48-4.17)	**0.001**	1.82 (1.12-2.94)	**0.015**
aOR (95% CI)	Reference	1.25 (0.78-1.99)	0.360	2.23 (1.30-3.83)	**0.004**	1.32 (0.79-2.20)	0.268
LBR							
OR (95% CI)	Reference	0.65 (0.52-0.81)	**0.000**	0.73 (0.54-0.98)	**0.036**	0.63 (0.49-0.81)	**0.000**
aOR (95% CI)	Reference	0.72 (0.57-0.91)	**0.006**	0.76 (0.55-1.04)	0.087	0.74 (0.57-0.96)	**0.022**

CPR, clinical pregnancy rate; AR, abortion rate; LBR, live birth rate; OR, Ratio ratio; aOR, Adjusted Ratio ratio; bold values means P < 0.05 was considered to be statistically significant.

As visible in **Table 3A**, female and/or male BMI had influence on CRP, AR, and LBR not only in the IVF cycles, but also in the ICSI cycles. After the confounding factors were excluded by logistic regression analysis, we found that female and male BMI affected mainly AR and LBR, but not CPR in the IVF cycles. Compared with Group I (the reference), the AR in Group IV was higher (aOR: 1.46, 95% CI: 1.17–1.81, P = 0.000), whereas the LBR was lower (aOR: 0.86, 95% CI: 0.75–0.97, P = 0.017) (**Table 4A**). However, a difference was present between the ICSI cycles and IVF cycles. Specifically, in the ICSI cycles, CPR in Group ii was significantly lower than that in Group i after excluding the confounding factors (aOR: 0.73, 95% CI: 0.58–0.93, P = 0.012) (**Table 4B**). The value of AR in Group iii was considerably higher than that in Group I (aOR: 2.23, 95% CI: 1.30–3.83, P = 0.004). LBR in Group ii and Group iv were significantly lower (aOR: 0.72, 95% CI: 0.57–0.91, P = 0.006; aOR: 0.74, 95% CI: 0.57–0.96, P = 0.022) than that in Group i (**Table 4B**).

## Discussion

Following social development, the living standard of human beings has been substantially improved, which changed lifestyle. The proportion of obese people worldwide has been steadily increasing. Over recent years, the effect of obesity on egg and sperm quality and the effect of obesity on embryo development, pregnancy, and obstetric outcomes have attracted significant research attention in the field of assisted reproduction. Although many studies have been published, the findings on the effects of BMI on oocytes and sperms quality, embryo development, and the ART process outcomes remain controversial.

In our study, we found differences in female’s and male’s age in different BMI subgroups. This was largely due to personal habits and lifestyle variety. We also found differences in the levels of FSH, LH, AMH, and other hormones in the women in different BMI groups. These variations might have been caused by pulses of progonadoliberin-1 produced by the hypothalamus, which stimulates the secretion of follicle-stimulating hormone (FSH) and luteinizing hormone (LH) by the pituitary. Then, FSH and LH induce the secretion of steroid hormone by the ovarian and testicular tissue, leading to excessive androgen production in women and estrogen production in men. The elevated concentrations of androgen and estrogen exert a negative feedback effect on the hypothalamus-pituitary-ovary/testis axis. This could affect gonadotropin production, causing fluctuations and even imbalances in the reproductive hormones (43, 44). The results of our baseline data analysis showed that the AFC, No. of oocytes retrieved, and No. of MII oocytes in overweight and obese women were higher than those in normal weight women, which may be related to the high proportion of overweight and obese women with PCOS. We found that men with higher BMI had lower semen volume and PR, but had higher NP, IM and DFI%. Sperm movement is produced by adenosine triphosphate (ATP) continuously produced by mitochondria located in the middle of the sperm. A higher BMI leaded to overproduction of reactive oxygen species (ROS). An excess of ROS alters the phospholipid membrane, thereby destroying its selectivity, and also inhibits oxidative phosphorylation, ultimately resulting in reduced ATP production. And the ROS overproduction resulting in the oxidation of DNA bases (mainly guanosine) by producing by-products of lipid degradation that bind to DNA or come in direct interaction with DNA strands, resulting in nonspecific C single and double-strand breaks and increased DNA damage (45–49).

The accumulation of adipose tissue was found that could to lead to oxidative stress and DNA damage (e.g., DNA methylation), leading to defective oocytes and embryo development in offspring mice (50–52). Early embryonic development is known to be driven mainly by oocytes. Han et al. found that the absence of Stella protein (also known as DPPA3 or PGC7) in the oocytes of obese female mice could mediate epigenetic effects such as hydroxymethylcytosine modification and DNA damage (53). Triglyceride was established to be lipotoxic to embryos, and that palmitic acid, the most common fatty acid in human serum, could cause low proliferation of embryonic trophoblast stem cells and dose-dependent apoptosis (54). Our results showed that compared with normal female, the high-quality embryo rate in the female subgroups with BMI ≥ 24 kg/m² were lower in the ICSI cycles but not in the IVF cycles, which was consistent with the results of Depalo et al. (14). Another study showed that obesity in women resulted in poor IVF outcomes but did not affect embryo quality (55). However, this study was retrospective and did not eliminate enough confounding factors. Meanwhile, male BMI had no effect on high-quality embryo rate when the female BMI was normal. At female BMI ≥ 24 kg/m², the high-quality embryo rate in the male BMI ≥ 24 kg/m² subgroup was lower than that in the male BMI ≤ 24 kg/m² subgroup, but the difference was not statistically significant. This result was consistent with the findings of Anifandis et al. (32).

A systematic review conducted in 2021 suggested that a statistical association was found between female higher BMI and lower CPR, higher AR and lower LBR (15). In our study, we found that only a high female BMI did not negatively affect CPR not only in IVF cycles but also in ICSI cycles. We found low CPR in male with high BMI regardless of the female BMI in the ICSI cycles. However, CPR was affected only if the female had a high BMI and the male had an abnormal BMI in the IVF cycle. After adjustments for confounding factors, we also revealed that neither female nor male BMI exerted any effect on CPR in the IVF cycles. In our study, we found no effect of female BMI on CRP, which was consistent with the conclusions of Haghighi et al. (26). After adjustments for confounding factors, we found that only high female and male BMI resulted in high AR and low LBR in IVF. Meanwhile, high female BMI individually led to high AR in ICSI when the male’s BMI was normal. In the ICSI cycles, LBR in Group ii and Group iv were lower than that in the reference group. The male’s BMI had a greater impact on LBR than the female’s BMI. This might because of female and male with higher BMI might affect the quality of oocytes and sperms, and thus the development and quality of the embryo, which in turn affects CPR, LBR, and AR.

In our study, we conducted multiple stratified analyses to explore the effects of BMI in both men and women on the embryo development and ART outcomes. IVF and ICSI cycle analyses were conducted. We analyzed the effects of BMI of men and women on oocyte, semen quality, embryo development, embryo quality, and ART outcomes, but we lacked basic research on the mechanisms underlying these effects. This is something we need to look at further.

## Conclusion

In conclusion, the effects of female and male BMI on embryo development and ART outcomes remain controversial. Thus, to improve the quality of assisted reproduction, more prospective and basic research is needed to explore the impact of BMI on human fertility, which was the purpose of this investigation. In the ICSI cycle, an abnormally higher female BMI was not conducive to the formation of high-quality embryos. Female and/or male BMI affected AR and LBR more than CPR not only in the IVF cycles but also in the ICSI cycles. However, our study had limitations as it was retrospective. Therefore, further research is needed to confirm our findings.

## Data Availability Statement

The original contributions presented in the study are included in the article/supplementary material. Further inquiries can be directed to the corresponding author.

## Ethics Statement

Written informed consent was obtained from the individual(s) for the publication of any potentially identifiable images or data included in this article.

## Author Contributions

LQ developed the original concept of this study. LQ, Y-PL and Y-CS participated in the study design. Y-PL, X-LC, S-MW and N-NW participated in data collection, Y-PL conducted data analysis and interpretation and the writing of the original version of the manuscript. HS guided the data analysis and gave guidance. All authors participated in the manuscript revision. All authors have contributed to critical discussion and reviewed the final version.

## Funding

This work was supported by the Chinese Natural Science Funds Youth Funds (81701505).

## Conflict of Interest

The authors declare that the research was conducted in the absence of any commercial or financial relationships that could be construed as a potential conflict of interest.

## Publisher’s Note

All claims expressed in this article are solely those of the authors and do not necessarily represent those of their affiliated organizations, or those of the publisher, the editors and the reviewers. Any product that may be evaluated in this article, or claim that may be made by its manufacturer, is not guaranteed or endorsed by the publisher.
